# Evaluating the Performance of Lateral Flow Devices for Total Aflatoxins with Special Emphasis on Their Robustness under Sub-Saharan Conditions

**DOI:** 10.3390/toxins13110742

**Published:** 2021-10-20

**Authors:** Barbara Cvak, Benedikt Warth, Joseph Atehnkeng, Alexandra Parich, Alexandra Moritz, Michael Sulyok, Rudolf Krska

**Affiliations:** 1 University of Natural Resources and Life Sciences, Vienna, Department of Agrobiotechnology, IFA-Tulln, Institute of Bioanalytics and Agro-Metabolomics, Konrad-Lorenz-Strasse 20, 3430 Tulln an der Donau, Austria; barbara.cvak@dsm.com (B.C.); alexandra.parich@boku.ac.at (A.P.); michael.sulyok@boku.ac.at (M.S.); 2 Romer Labs Division Holding GmbH, Technopark 5, 3430 Tulln an der Donau, Austria; 3 Department of Food Chemistry and Toxicology, University of Vienna, Währinger Straße 38, 1090 Vienna, Austria; benedikt.warth@univie.ac.at; 4 International Institute of Tropical Agriculture (IITA), Ibadan 200001, Nigeria; j.atehnkeng@cgiar.org; 5 EV Group E. Thallner GmbH, DI Erich Thallner Strasse 1, 4782 St. Florian am Inn, Austria; a.moritz@EVGroup.com; 6 Queen’s University Belfast, School of Biological Sciences, Institute for Global Food Security, University Road, Belfast BT7 1NN, Northern Ireland, UK

**Keywords:** lateral flow devices (LFDs), lateral flow immunoassays, strip tests, mycotoxins, aflatoxins, food safety

## Abstract

As aflatoxins are a global risk for humans and animals, testing methods for rapid on-site screening are increasingly needed alongside the standard analytical laboratory tools. In the presented study, lateral flow devices (LFDs) for rapid total aflatoxin screening were thoroughly investigated with respect to their matrix effects, cross-reactivity, their performance under harsh conditions in Sub-Saharan Africa (SSA), and their stability, as well as when compared with liquid chromatography-tandem mass spectrometry (LC-MS/MS). To analyze the matrix effects, qualitative test kits offering a certain cutoff level were used to screen different nut samples. In addition, these tests were challenged on their cross-reactivity with 230 fungal toxins and metabolites. Furthermore, the resulting measurements performed under harsh tropical conditions (up to 38.4 °C and 91% relative humidity) in SSA, specifically Burkina Faso and Mozambique, were compared with the results from a well-established and validated LC-MS/MS-based reference method. The comparison of the on-site LFD results with the reference method showed a good agreement: 86.4% agreement, 11.8% non-agreement, and 1.8% invalid test results. To test the robustness of the cutoff tests, short- and long-term stability testing was carried out in Mozambique and Nigeria. For both experiments, no loss of test performance could be determined. Finally, a subset of African corn samples was shipped to Austria and analyzed under laboratory conditions using semiquantitative aflatoxin tests. A good correlation was found between the rapid strip tests and the LC-MS/MS reference method. Overall, the evaluated LFDs showed satisfying results regarding their cross-reactivity, matrix effects, stability, and robustness.

## 1. Introduction

Apart from the classical analytical methods based on chromatographic techniques, fast and inexpensive on-site tools without the time-consuming sample preparation and cleanup steps are needed for the screening of mycotoxins in susceptible crops. The interest in rapid membrane-based immunoassay methods such as immune sensors, flow-through immunoassays, and lateral flow devices (LFDs) [1,2,3] has strongly increased due to the need for rapid on-site (pre) screening. Requiring no sample preparation other than grinding and extraction, LFDs, also named strip tests, allow for the qualitative or semiquantitative determination of mycotoxins within a few minutes. The strong interest in this approach is reflected in the increasing number of commercially available test kits for field use based on direct competitive assays.

LFDs are available for all mycotoxins regulated in the European Union; however, aflatoxins are the most important mycotoxin group due to their toxicity and occurrence [4,5,6]. Aflatoxins are mainly produced by the fungi *Aspergillus flavus* and *Aspergillus parasiticus* [7] and are toxic and carcinogenic [8]. There are four principal types of these toxins in contaminated plant products: B1, B2, G1, and G2. Of these, aflatoxin B1 is the most widely distributed and exhibits the highest toxicity. It causes liver disease in animals, is a potent carcinogen in humans [9], and can have other negative effects on the nervous, gastrointestinal, and renal systems. The production of aflatoxins on grain and other food and feedstuff strongly depends on both commodity and climatic conditions, as well as the storage conditions after the harvest. Africans, especially those populations living in Sub-Saharan Africa (SSA), are at a high risk for chronic dietary mycotoxin exposure due to consumption, especially since a large portion of the crops in tropical and subtropical regions are highly susceptible to mycotoxin contamination [10,11]. 

As a result, regulatory authorities have set limits for food and feed. The European Commission has adopted maximum limits for aflatoxins in food and feedstuff ((EC) No 165/2010) [12] as well as for groundnuts (peanuts) and other oilseeds, tree nuts, apricot kernels, licorice, and vegetable oil ((EC) No 178/2010) [13], while in the United States there are action levels set to monitor mycotoxin contamination [14]. However, the regulatory limits in SSA are still partially lacking or improperly implemented; thus, the surveillance of mycotoxin contamination is still a major issue. This is especially true for food intended for consumption by local populations.

For example, Burkina Faso has not yet implemented any mycotoxin regulations. By contrast, Mozambique has defined a maximum limit of 10 µg/kg total aflatoxins (the sum of aflatoxin B1, B2, G1, and G2) for corn, peanuts, peanut butter, peanut milk, cereals, and feedstuffs according to a survey of worldwide mycotoxin regulation performed by the Food and Agriculture Organization (FAO) in 2003 [15]. 

In many African countries, the standard analytical methods for mycotoxin analysis such as HPLC and LC-MS/MS are not accessible due to a lack of adequate equipment, the accessibility of necessary liquid nitrogen, spare parts, technicians, and/or laboratory staff with limited training 

However, the aflatoxin contamination in grains and other commodities like peanuts is still critical. Within the past few decades, several outbreaks have been reported in different, mainly Sub-Saharan, countries [16,17,18,19].

More recently, the situation in Africa was described in detail by Meijer et al. [20], combining data from the entire continent within a systematic literature review covering the period from 2010 to 2018. In their findings, the mean aflatoxin B1 concentration in maize exceeded the European Union legal limit, thereby resulting in a high overall exposure that is causing an increase in long-term disease.

Therefore, the demand for additional methods, such as simple strip tests for the on-site screening of mycotoxins, is increasing. 

The present study describes the evaluation of commercially available qualitative LFDs for total aflatoxins with respect to their matrix effects and cross-reactivity. Furthermore, the qualitative LFDs were challenged by using the tests on-site under harsh tropical conditions, and their results were compared to a well-established LC-MS/MS reference method [21]. Moreover, the tests were investigated with respect to their stability when stored under extreme climatic conditions (i.e., high temperature and humidity). 

Additionally, 31 corn samples from Burkina Faso and Mozambique were shipped to Austria and investigated using semiquantitative strip tests. The obtained results were also compared with the LC-MS/MS reference method.

## 2. Results & Discussion

### 2.1. Matrix Effect Testing 

Each matrix was spiked with the aflatoxin standard in triplicate, and each extract was analyzed in duplicate. All the results at a single level, as well as all the replicates, showed similar results, and the summarized results are given in Table 1. 

The cutoff levels (Section 4.1) of the evaluated matrices were slightly different from the expected value; however, no false negative results were obtained. It can be assumed that the matrix type has a slight influence on the cutoff values of the rapid tests that were investigated. Peeled almonds and peanuts showed a positive result at the cutoff level, whereas the unpeeled nuts showed a positive result slightly above the cutoff level in the test that was used. The work from Zhang et al. analyzed the aflatoxins in peanuts and other commodities [22,23] and reported good recoveries when testing with different matrices such as nut samples. 

Overall, the study of these commercially available test kits for aflatoxins confirmed their applicability for the qualitative determination of the selected matrices as stated by the manufacturer. 

Further experiments were carried out using ethanol instead of methanol as an extraction solvent. Both test kits with cutoff values of 4 µg/kg and 20 µg/kg, respectively, were validated using the spiked peeled peanut samples. This experiment confirmed that toxic methanol may be substituted by ethanol, as the obtained results were valid at all fortification levels when using ethanol. As the test results for the ethanol and methanol extracted samples were similar, detailed data has not been provided. 

### 2.2. Cross-Reactivity Testing

Cross-reactivity studies for the immune-based rapid tests for aflatoxins are done to determine the potential cross-reactivity against the different types of aflatoxins (i.e., AfB1, AfG1, AfB2, AfG2, and AfM1), as described by Santos et al. [24], or against other mycotoxins (i.e., deoxynivalenol, zearalenone, ochratoxin, and, fumonisins), as previously outlined by Zhang et al. [22]. To the best of our knowledge, the cross-reactivity of the aflatoxin lateral flow devices have not been investigated to the extent presented in this paper. 

The mix standards (mix 1–mix 23, given in Section 4.3) were diluted and used as samples to check the LFDs (test kit A) for cross-reactivity against any of the contaminating substances that were present. The test procedure was performed according to the manufacturer’s instructions. The presence of a visible test line indicated a test result below the threshold, as explained in detail in Section 4.1. This was true for all the mixes. In the case of Mix 5, 15, 17, 18, and 21, only faint lines were observed. For this reason, all the compounds included in those mixes were evaluated as single compounds and were subsequently found to be negative (a visible line appeared), with the exception of four substances: citreoviridin, mithramycin, K252a, and puromycin. In these four instances, very faint lines could still be observed. A possible reason for the variation of the line intensity may be related to the tested substances, or may be a result of the variation in the test kit production; however, the results were stated to be “not relevant” because the results can only be considered positive when no visible test line is obtained. Therefore, the qualitative cutoff tests were found not to be cross-reactive against any of the substances and only demonstrated reactivity against total aflatoxins (AfB1, AfB2, AfG1, and AfG2). 

### 2.3. On-Site Testing under Sub-Saharan Conditions in Burkina Faso and Mozambique

Rapid tests such as the lateral flow devices have previously been used for on-site mycotoxin detection, as reported by, e.g., Xu et al. [25], where the results for maize and feed samples were in excellent agreement with those from the high-performance liquid chromatography–tandem mass spectrometry. 

In this study, a total of 110 samples collected in Burkina Faso and Mozambique were analyzed using the qualitative strip tests under extreme weather conditions. We had the opportunity to collect a vast number of different samples, including several variations of corn, cornflakes, couscous, feed, groundnut, infant food, millet, rice, sesame, sorghum, soy, and wheat, as described in detail in Table 2. 

Following the on-site analysis, all the samples were tightly sealed and shipped to Austria for analysis by LC-MS/MS, and the detailed results of both measurements are presented below.

The majority of the LFD results correlated with the selected reference method. Of the 110 samples analyzed, 95 samples (86.4%) were in agreement with the reference method while 13 samples (11.8%) indicated conflicting results and 2 readings (1.8%) were considered invalid. From the 13 misaligned results, four samples resulted in false negative results (3.6%) by the LFD, one sample indicated a false positive result (0.9%), and the remaining eight results were underestimations (7.3%), meaning the measured concentration by the LC-MS/MS was higher than the results obtained when using the strip tests. No correlation was found between the 13 misaligned results, as different matrices and variable origins of the samples were affected. 

The potential reasons for the few incorrect LFD results could either be due to spot contamination of the aflatoxin, as different subsamples were analyzed by the strip tests and the LC-MS/MS, or due to the grain size since the ground samples that were analyzed on-site were not as finely ground as those samples used for the laboratory analysis. The on-site samples were mostly ground using traditional mortars, while the samples for the LC-MS/MS analysis were ground using standard laboratory mills. In addition, the high temperatures during the analysis could also explain some of the conflicting results, as most of the tests were performed at temperatures higher than 30 °C. Additionally, the resulting extraction efficiency from shaking the samples by hand for 1 min could also prove to be critical. 

To conclude, the results of the LFDs were satisfactory considering that the tests were mainly performed under tropical conditions. An easy, fast, and inexpensive estimation of the contamination level of a sample could be acquired without the need for expensive lab equipment since the kit functions as a stand-alone product and does not require any additional equipment or reagents except for the solvent used for the extraction. A potential problem presented in this study was the supply and availability of the analytical solvents in rural areas. However, this also allowed for both methanol and the nontoxic ethanol to be evaluated for use as extraction solvents. 

A potential drawback may arise from the subjective interpretation of the results, since this remains operator dependent. When a very faint test line was visible, the interpretation of the results was critical because faint test lines have also been interpreted as negative. This may be the reason for some of the misaligned LFD results, especially where the LC-MS/MS provided positive results. 

To summarize, 86.4% of the LFD results aligned with the results obtained when using a high-end LC-MS/MS reference method. 

### 2.4. Stability Study on Qualitative Test Kits

To evaluate the robustness of the test kits, repeated measurements over a certain time period (i.e., stability studies) were carried out using test kit B (as described in Section 4.4).

Despite the harsh storage conditions, no decrease in the stability could be monitored for short-term stability testing. For all the negative controls, a visible line appeared in the test zone of the strip; for all positive controls, no lines were visible

**Figure 1:**
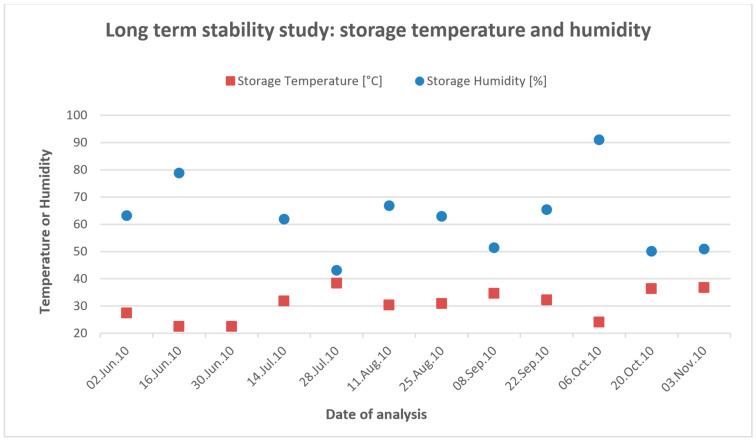
Long term stability study of 10 µg/kg cutoff LFD carried out in Nigeria for a time period of 5 months (June 2010–November 2010).

A long-term stability study was carried out in Nigeria by testing the negative controls (50% EtOH) and the positive controls (50% EtOH + 15 µg/kg AfB1) over a time period of 5 months. Tests of the same batch were stored both refrigerated (storage temperature 3.5–4.0 °C at 61.3–86.3% relative humidity) and at an ambient temperature, and were tested bi-weekly (all tests were performed in duplicate). For all the negative controls, a visible line appeared; for all the positive controls, no line was visible no matter at which temperature the test kits were stored. The details of the ambient storage conditions are given in Figure 1.

According to the results, the performance of the test kits did not deteriorate over a time period of 5 months regardless of the storage conditions (room temperature vs refrigerated), and thus the storage temperature does not appear to influence the test results. 

### 2.5. AgraStrip Aflatoxin Semiquantitative Test Kit

Of the 45 African corn samples, 31 of the samples (17 samples collected in Burkina Faso and 14 samples collected in Mozambique) were taken to be additionally analyzed using semiquantitative aflatoxin tests. The remaining 14 samples could not be analyzed due to an insufficient amount of sample availability (less than 30 g of sample). Each corn sample was extracted in triplicate, with each extract analyzed using the semiquantitative LFDs, and results were compared to the measurements from a well-established and fully in-house validated dilute-and-shoot LC-MS/MS method [21]. The detection range of the rapid test was 3–100 µg/kg, and within all the investigated samples neither false positive results nor false negative results were observed. 

**Figure 2:**
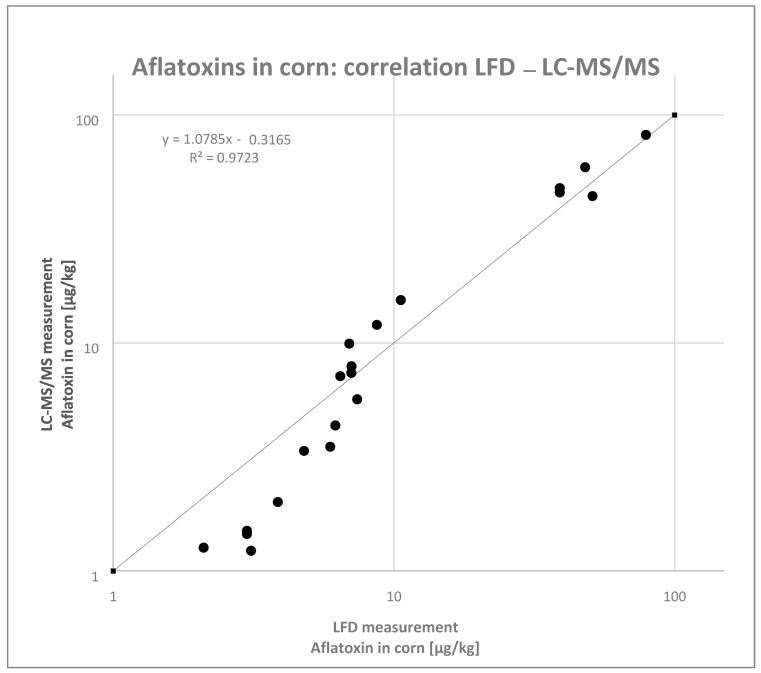
Aflatoxin concentration in corn. Semiquantitative LFD vs. LC-MS/MS.

In total, 18 of the 31 results were within the detection range of the rapid test and could therefore be compared to the reference method as given in Figure 2. 

Seven of the LFD results < LOQ could be verified by the LC-MS/MS (the LOD of AfB1 was 0.8 µg/kg) and six of the LC-MS/MS positive samples were found to be out of the LFD calibration range (LFD results > 100 µg/kg). The LFD results in the lower calibration range, especially below 10 µg/kg, were overestimated by the trend. However, the results of the 18 samples with results between 3–100 µg/kg indicated an acceptable correlation between both methods for the set of samples that were investigated in this study. The relative standard deviation (RSD) of all the measured samples, regardless of the method used, was below 20%. 

There was a good correlation between the test kit and the reference method when comparing the data obtained from both methods of analysis. 

It can be demonstrated that within a minimum of time and with manageable equipment the rapid method was able to provide reliable and satisfactory results when compared to a highly sophisticated analytical method, which had previously been reported [25]. 

When considering the rapid on-site monitoring, the quantitative strips can be used as a feasible alternative to the conventional lab methods. 

## 3. Conclusions

Lateral flow devices for the detection of aflatoxins were challenged with respect to their matrix effects, cross reactivity, and stability, as well as their robustness under harsh climatic conditions. 

The matrix effect was initially evaluated to determine the performance variance when different nut samples were used with qualitative tests that have a cutoff level of 4 µg/kg. The results were slightly different from the stated cutoff, depending on which kind of nut was being evaluated; however, none of the results were false negative or false positive. 

To evaluate the cross reactivity of the qualitative LFDs, over 200 different toxins and metabolites were evaluated. No cross-reactivity against any of the evaluated substances were found, which confirmed their specific reactivity only against aflatoxins. 

Furthermore, the performance of the qualitative cutoff tests were evaluated under tropical conditions in SSA at high temperatures and a high relative humidity. 

Over 100 samples, including several different kinds of grain, nuts, rice, and feed samples, were evaluated under these conditions in Burkina Faso and Mozambique. Moreover, the test strips were stressed by the storage temperature of the test kits. Despite the critical storage conditions at high ambient temperatures, their robustness was demonstrated under both short- and long-term stability studies. More than 86% of the results showed agreement with the results obtained using a laboratory reference method. Thirteen of the samples (11.8%) resulted in a disagreement with the LC-MS/MS reference values, which may be due to nonhomogeneous samples or the grain size. Additionally, a subset of the evaluated corn samples were shipped to Austria and evaluated under laboratory conditions using semiquantitative LFDs. The results were then compared with the results from the standardized LC-MS/MS reference method. Overall, the strip test results showed a good correlation in the range of 3–100 µg/kg aflatoxins in corn, and neither false positive nor false negative results were obtained. 

Both test kit versions, the qualitative and the semiquantitative strip tests, demonstrated satisfactory results and therefore provide a great alternative wherever the time and the costs of the analysis are crucial. The easy-to-use test strips are a good alternative to monitor mycotoxin contamination on-site, especially in parts of the world where highly sophisticated laboratories are rare. 

## 4. Materials and Methods

### 4.1. Chemicals and Reagents

The evaluated lateral flow devices and the semiquantitative strip tests were the AgraStrip^®^ Total Aflatoxin Test (cutoff levels 4 µg/kg, 10 µg/kg, and 20 µg/kg, respectively) (Romer Labs, Tulln, Austria). 

**Figure 3:**
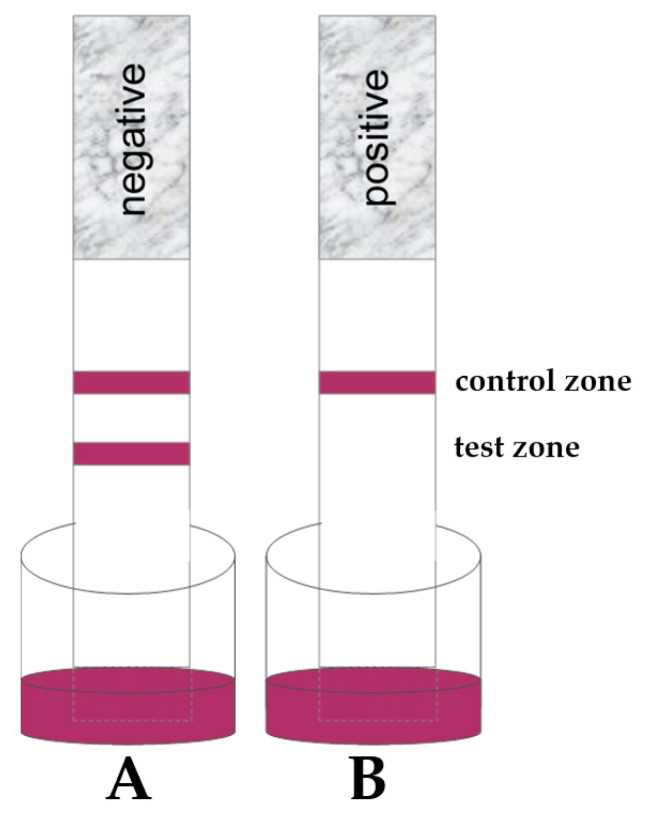
Negative (**A**) and positive (**B**) test results of qualitative competitive LFDs.

Mycotoxin strip tests are based on a competitive assay format, which means the analyte in the sample (aflatoxin) competes with bound aflatoxin on the test line. If no analyte is present in the sample, a line appears and indicates a negative test result. When an analyte is present in the sample, the competition will occur and at the given cutoff level the line disappears, which indicates a positive test result (as shown in Figure 3). Next to the test line, a second line in the control zone will always be visible to ensure the correct test development. When the control line is absent, the test result is considered invalid. 

According to the manufacturer’s package insert, the detection range of the semiquantitative test was 5–100 µg/kg. To get the quantitative results, strips were analyzed using the Romer Labs AgraVision^TM^ Reader. Extraction and test procedure was performed according to the package insert. In-lab testing was performed using methanol purchased from VWR (Radnor, PA, USA) while on-site testing (SSA) made use of methanol and ethanol provided locally.

### 4.2. Matrix Effect Testing

Peeled and unpeeled almonds, macadamia nuts, para nuts, peeled and unpeeled peanuts, and pecan nuts were purchased at a local Austrian market (Naschmarkt, Vienna). All seven samples were analyzed in triplicate by HPLC-MS/MS prior to matrix effect evaluation with the lateral flow devices. 

For the strip testing, 10 g of each ground sample was weighed out in triplicate and spiked with 0, 2, 3, 4, 5, and 6 µg aflatoxin B1 standard per kg matrix, respectively. Briefly, the liquid aflatoxin B1 standard solution in acetonitrile (provided by Romer Labs, Tulln, Austria) was dispensed onto the top of the ground sample and the solvent was allowed to evaporate for 30 min at room temperature. The spiked samples were extracted in a ratio of 1:2 (10 g sample + 20 mL extraction solvent) using 70% MeOH (MeOH–H_2_O, 70:30) and shaken by hand for 1 min. The sample was allowed to settle for 1 min, the supernatant was removed, and then subsequently used for analysis by following the manufacturer’s test kit instructions. Each extract was analyzed in duplicate. 

Furthermore, peeled and unpeeled peanut samples were additionally tested by using 50% EtOH (EtOH–H_2_O/50:50) instead of the 70% MeOH for the extraction so to test if methanol could be replaced by the less harmful ethanol, as stated in the manufacturer’s manual. Except for extraction solvent, all the steps were done in accordance to the previously described method. 

### 4.3. Cross-Reactivity Testing

The cross-reactivity of the strip tests for the aflatoxins was investigated with the mixtures of the liquid standards used for the multi-mycotoxin analysis. The sources of these liquid standards of approximately 230 fungal toxins and metabolites are given in reference [26]. 

In total, 23 mixtures of mycotoxins and metabolites were used in this study. The composition of each mix is provided in Table 3.

The solutions were stored at −20 °C and allowed to reach room temperature, unassisted, prior to use. All mixes were diluted to 1:20 using 70% MeOH (MeOH–H_2_O, 70:30) before testing. The final concentrations of the evaluated solutions were much higher than the given cutoff level of the used test kit (4 µg/kg, test kit A, as described in Section 4.4). Therefore, clear results could be expected. 

### 4.4. On-Site Testing in Burkina Faso and Mozambique

For extensive on-site evaluation, three aflatoxin tests were used (as given in Table 4). These tests are qualitative tests with a given cutoff level, meaning the indication of a negative or positive test result at a certain concentration level (as shown in Figure 3). 

For clarity, when a sample was analyzed with test kit A and no test line was visible after the stipulated run time, it indicated an aflatoxin concentration in the sample higher than 4 µg/kg (4 µg/kg is the cutoff level of test kit A). 

For test kits B and C, the cutoff levels are 10 µg/kg and 20 µg/kg, respectively, following the same principle: when the analyzed concentration of aflatoxin is higher than the cutoff level of the used test, no test line is expected in the test zone. 

The analyzed samples were obtained from several different locations in Burkina Faso and Mozambique, as previously described by Warth et al. [27]. From those 122 samples, 110 samples (as given in Table 2) were tested on-site for their aflatoxin contamination by using qualitative LFDs. In Burkina Faso, the extraction was done with 50% ethanol (EtOH–H_2_O, 50:50) while in Mozambique 70% methanol (MeOH–H_2_O, 70:30) was applied. All extracts were initially tested using test kit A. All the samples indicating a positive result were further evaluated using test kit B and test kit C to estimate the contamination level. 

All the tests were performed on-site, whereby some measurements were carried out inside and some outside of a building; however, typically the measurements were done in a barn or under a tree with temperatures up to 38.4 °C and a relative humidity up to 91%. 

In order to verify the analytical performance of the LFD test kit, all the samples were shipped to Austria and promptly measured by a well-established LC-MS/MS method for the multi-mycotoxin analysis as described by Sulyok et al. [21]. 

### 4.5. Stability Study on the Qualitative Test Kit

Short-term stability testing was done in Nampula province, Mozambique over a one-week period, where the test kits were continuously stored under ambient conditions and partly in direct sunlight (with a temperature of at least 25 °C and relative humidity of approximately 35%). A fresh positive control, 15 µg/kg aflatoxin B1 standard in 70% MeOH (MeOH–H_2_O, 70:30), and a negative control, 70% MeOH (MeOH–H_2_O, 70:30) only, were prepared daily and measured in duplicate. 

Furthermore, a long-term stability study was carried out in Ibadan, Nigeria. Strips from test kit B were tested bi-weekly over a period of 5 months. Kits from the same batch were stored at 4 °C and at ambient temperature in darkness (without air conditioning). As no MeOH was available in Nigeria, 50% EtOH (EtOH–H_2_O, 50:50) was used instead for the extraction.

For each measurement, a positive control of 15 µg/kg aflatoxin B1 standard in 50% EtOH (EtOH–H_2_O, 50:50), and a negative control of only 70% MeOH (MeOH–H_2_O, 70:30) were analyzed using the test kits stored at both temperatures. Standard solutions were freshly prepared for each measurement and the cooled kit was allowed to climatize to room temperature prior to use. Temperature and humidity were monitored during testing. 

### 4.6. Quantitative Total Aflatoxin Test Kit

Each of the 31 corn samples were extracted in triplicate. Briefly, 10 g of each sample were weighed in triplicate and extracted in a ratio of 1:2, with 10 g of ground sample + 20 mL of 70% MeOH (MeOH–H_2_O, 70:30), and with 1 min of shaking by hand. Extracts were allowed to settle for 2 min 30 sec and were further diluted to 1:20 (ratio of 1 + 19, i.e., 50 µL sample extract + 950 µL dilution buffer) using the dilution buffer provided in the test kit. The procedure was precisely followed as stated in the manufacturer’s package insert. For the analysis, 100 µL of each extract was pipetted into a small microwell, then a strip was inserted into each well and allowed to develop for 3 min. The test was performed at a constant temperature (35 °C) by using the heat block provided by the manufacturer. The intensity of the emerging color test line was analyzed using the AgraVision^TM^ Reader to obtain the quantitative results. The LFD results were compared with the results gained by the established LC-MS/MS reference method by Sulyok et al. [21] using an AB Sciex 4000 QTRAP^®^ system. 

## Figures and Tables

**Table 1:** Matrix effect of 4 µg/kg cutoff aflatoxin test, *n* = 6 (number of replicates).

Spiking Level	Blank	2 µg/kg	3 µg/kg	4 µg/kg	5 µg/kg	6 µg/kg
Almonds unpeeled	-	-	-	-	-	+
Almonds peeled	-	-	-	+	+	+
Macadamia nuts	-	-	~	+	+	+
Para nuts	-	-	-	~	~	+
Peanuts unpeeled	-	-	-	-	+	+
Peanuts peeled	-	-	-	+	+	+
Pecan nuts	-	~	+	+	+	+

+ positive result; - negative result; ~ result unclear (very faint line visible).

**Table 2:** Comparison of qualitative LFD results (gained on-site) with quantitative LC-MS/MS results. BF—Burkina Faso; M—Mozambique; INERA—the Institute of Environment and Agricultural Research; CTRAPA—the Centrale de Transformation de Produits Agricoles; LNSP—the Laboratoire National de Santé Publique; not valid—incorrect LFD result due to missing control line; nd—not detected; all values given in µg/kg.

Sample Number	Matrix	Country of Origin	Location/Origin	Result LFD Onsite Testing	LC-MS/MS Data Expressed as LFD Comparison	Results LC-MS/MS	LFD Results Are in Line with Reference Method
1	cornflakes	BF	INERA	not valid	<4	nd *	-
2	cornflakes	BF	INERA	<4	<4	nd	yes
3	cuscus corn	BF	CTRAPA	<4	4–10	5	no
4	cuscus mix	BF	CTRAPA	<4	<4	nd	yes
5	cuscus mix	BF	CTRAPA	<4	<4	nd	yes
6	cuscus rice	BF	CTRAPA	<4	<4	nd	yes
7	feed corn	BF	CTRAPA	>20	>20	674	yes
8	feed corn	BF	CTRAPA	>20	>20	649	yes
9	feed product	M	Rapale	10–20	>20	57	no
10	feed product	M	Guttlimidada	<4	<4	nd	yes
11	groundnut	BF	Ouaga market	<4	<4	nd	yes
12	groundnut	BF	Nagreongon	<4	<4	nd	yes
13	groundnut	BF	Boromo	<10	<4	nd	yes
14	groundnut	BF	Boromo	<10	<4	nd	yes
15	groundnut	BF	INERA	<4	<4	nd	yes
16	groundnut	BF	INERA	4–10	10–20	16	no
17	groundnut	BF	INERA	<4	4–10	6	no
18	groundnut	BF	INERA	<4	<4	nd	yes
19	groundnut	BF	INERA	<4	<4	nd	yes
20	groundnut	M	Nampula province	>20	>20	173	yes
21	groundnut	M	Nampula province	<4	<4	nd	yes
22	groundnut	M	Nampula province	>20	<4	nd	no
23	groundnut	M	Nampula province	<4	<4	nd	yes
24	groundnut	M	Nampula province	<4	<4	nd	yes
25	groundnut	M	Nampula province	<4	<4	nd	yes
26	groundnut	M	Nampula province	<4	<4	nd	yes
27	groundnut	M	Nampula market	>20	>20	643	yes
28	groundnut	M	Ikuru	<4	<4	nd	yes
29	groundnut	M	Nampula province	<4	<4	nd	yes
30	groundnut	M	Nampula province	<4	<4	nd	yes
31	groundnut	M	Nampula province	<4	<4	nd	yes
32	groundnut	M	Nampula province	<4	<4	nd	yes
33	groundnut	M	Nampula province	<4	<4	nd	yes
34	groundnut	M	Nampula province	<4	<4	nd	yes
35	groundnut	M	Nampula province	<4	<4	nd	yes
36	groundnut	M	Netia	<4	<4	nd	yes
37	groundnut	M	Nacololo	<4	<4	nd	yes
38	groundnut	M	Nacololo	<4	<4	nd	yes
39	groundnut	M	Namitil	<4	<4	nd	yes
40	groundnut feed	BF	Bobo	>20	>20	110	yes
41	groundnut feed	M	Ikuru	>20	>20	185	yes
42	groundnut seed	M	Ikuru	<4	<4	3	yes
43	infant food	BF	Ouaga, DTA	<4	<4	nd	yes
44	corn	BF	Quaga MELS	>20	>20	30	yes
45	corn	BF	Nagreongon	<4	<4	nd	yes
46	corn bran	M	Said Agro Industria	>20	>20	471	yes
47	corn bran	M	Guttlimidada	>20	>20	311	yes
48	corn feed	M	Cimpan Lda	<4	<4	nd	yes
49	corn feed	M	Ikuru	>20	>20	135	yes
50	corn feed	M	Said Agro Industria	>20	>20	482	yes
51	corn flour	BF	Sitrac	>20	>20	48	yes
52	corn flour	M	Cimpan Lda	<4	<4	nd	yes
53	corn flour	M	Said Agro Industria	~10	>20	49	no
54	corn flour	M	Said Agro Industria	~10	>20	41	no
55	corn flour fine	BF	Sitrac	>20	>20	126	yes
56	corn grain	BF	Sitrac	4–10	>20	44	no
57	corn seed	M	Ikuru	<4	<4	nd	yes
58	corn waste	M	Said Agro Industria	>20	>20	909	yes
59	corn white	BF	Ouaga market	<4	<4	nd	yes
60	corn white	BF	Boromo	<10	<4	nd	yes
61	corn white	BF	Boromo	<10	<4	nd	yes
62	corn white	BF	Boromo	<10	<4	nd	yes
63	corn white	BF	Ouaga, DTA	<4	<4	nd	yes
64	corn white	BF	Sodepal	not valid	4–10	7	-
65	corn white	BF	Velegda	>20	>20	682	yes
66	corn white	BF	Sitrac	<4	<4	nd	yes
67	corn white	M	Nampula market	>20	>20	81	yes
68	corn white	M	Nampula market	<4	<4	nd	yes
69	corn white	M	Cimpan Lda	<4	<4	nd	yes
70	corn white	M	Netia	<4	<4	nd	yes
71	corn white	M	Nacololo	<4	<4	nd	yes
72	corn white	M	Namitil	>20	>20	442	yes
73	corn white	M	Namitil	>20	>20	264	yes
74	corn white	M	Rapale	>20	>20	414	yes
75	corn white	M	Guttlimidada	<4	<4	nd	yes
76	corn white feed	BF	Bobo	>20	>20	881	yes
77	corn yellow	BF	Ouaga market	>20	>20	79	yes
78	corn yellow	BF	Quaga MELS	<4	<4	nd	yes
79	corn yellow	BF	Boromo	<10	<4	3	yes
80	corn yellow	BF	Ouaga, DTA	<4	<4	nd	yes
81	corn yellow	BF	Sitrac	<4	<4	nd	yes
82	corn yellow	BF	Sitrac	<4	<4	4	yes
83	corn yellow	BF	INERA	<4	<4	nd	yes
84	corn yellow	BF	CTRAPA	4–10	>20	99	no
85	corn yellow	BF	CTRAPA	<4	<4	nd	yes
86	corn yellow	BF	CTRAPA	4–10	>20	84	no
87	corn yellow	BF	CTRAPA	4–10	>20	60	no
88	millet	BF	Ouaga market	<4	<4	nd	yes
89	millet	BF	Sodepal	<4	<4	nd	yes
90	millet	BF	INERA	<4	<4	nd	yes
91	millet	M	Namitil	<4	<4	4	yes
92	millet	M	Rapale	<4	<4	nd	yes
93	rice Thai	BF	LNSP	<4	4–10	8	no
94	rice Thai	BF	LNSP	<4	<4	nd	yes
95	sesame	BF	Ouaga, DTA	<4	<4	nd	yes
96	sesame processed	BF	Boromo	<10	<4	nd	yes
97	sorghum	BF	Velegda	<4	<4	nd	yes
98	sorghum	BF	Boromo	<10	<4	nd	yes
99	sorghum	BF	Boromo	<10	<4	nd	yes
100	sorghum red	BF	Ouaga market	<4	<4	nd	yes
101	sorghum red	BF	INERA	<4	<4	nd	yes
102	sorghum white	BF	Ouaga, DTA	<4	10–20	16	no
103	sorghum white	BF	INERA	<4	<4	nd	yes
104	soy defatted	M	Rapale	<4	<4	nd	yes
105	soy full fat	M	Rapale	<4	<4	nd	yes
106	soy full fat	M	Guttlimidada	<4	<4	nd	yes
107	waste product	M	Rapale	4–10	4–10	5	yes
108	wheat	BF	Sodepal	<4	<4	nd	yes
109	wheat bran	M	Rapale	<8	<4	nd	yes
110	wheat bran	M	Guttlimidada	<4	<4	nd	yes

* Result < LOD. LOD Aflatoxins LC-MS/MS method: B1: 0.8 µg/kg; B2: 0.7 µg/kg; G1: 0.5 µg/kg; G2: 1 µg/kg.

**Table 3:** List of substances included in the determination of cross-reactivity; concentration (conc.) of each tested standard in mg/kg.

Substances [MIX 1]	conc.	Substances [MIX 2]	conc.	Substances [MIX 3]	conc.	Substances [MIX 4]	conc.
Sterigmatocystin	5	Chaetoglobosin A	5	Ergosin	2	Elymoclavin-Fructoside	2.2
Alternariol	5	Verruculogen	10	Ergotamin	2	Festuclavin	50
Penitrem A	7.5	Chetomin	15	Ergocristine	2	Iso-Dihydrolysergol	50
Emodin	5	Meleagrin	5	Ergocornin	2	Agroclavin	50
Alternariol	5	Verrucarin	2.5	Ergocryptin	2	Lysergol	5.2
Monomethylether		Cyclopiazonic acid	10	Ergometrin	4	Ox-Luol	50
Mycophenolsäure	14	Kojic acid	45	Dihydroergotamin	2	Elymoclavin	1.7
Citrinin	15	3-Nitropropionic acid	12.5	Dihydroergosin	0.4	Dihydrolysergol	9.9
Roridin A	8	Penicillic acid	10	Ergine	2	Ox-Elymoclavin	18.6
Tentoxin	2			Dihydroergin	2	Chancoclavin	2
Altenuen	5			Ergovaline	2		
**Substances [MIX 5]**	**conc.**	**Substances [MIX 6]**	**conc.**	**Substances [MIX 7]**	**conc.**	**Substances [MIX 8]**	**conc.**
Enniatin A	0.1	Physcion	10	Secalonic acid D	41.2	Cyclosporin C	40
Ennatin A1	0.4	Altenusin	10	Austocystin A	11.8	Cyclosporin D	40
Enniatin B	0.4	Aflatoxin M2	1	Viomellein	11.8	Cyclosporin H	40
Enniatin B1	1.1	Wortmannin	3.1	Apicidin	0.7	Macrosporin	30
Beauvericin	1	Fumagillin	2.5	Altertoxin I	47.1	Altersolanol	40
Enniatin B2	0.5	Pseurotin A	5	Aurofusarin	0.5		
Ennatin B3	1	Asterric acid	5	**Substances [MIX 11]**	**conc.**	**Substances [MIX 12]**	**conc.**
Enniatin B4	0.5	Cyclosporin A	5	Verrucofortine	10	Cytochalasin A	10
Enniatin J1	1	Fumigaclavin	5	Cyclopenin	20	Cytochalasin B	10
Enniatin K2	0.2			Paraherquamide A	20	Cytochalasin C	10
**Substances [MIX 9]**	**conc.**	**Substances [MIX 10]**	**conc.**	Pestalotin	20	Cytochalasin D	10
Pentoxyfylline	10	Monoacetoxyscirpenol	3.2	Phomopsin A	40	Cytochalasin J	10
Rubellin D	10	alpha-ZOL	3.2	Setusosin	20	Cytochalasin H	10
Cochliodinol	10	beta-ZOL	3.2	Mevastatin	20	HC-Toxin	10
Chaetocin	20	alpha-ZOL-Glucoside	6.3	Ophiobolin A	20	Brefeldin A	10
Tryprostatin A	20	beta-ZOL-Glucoside	6.3	3-Methylsterigmatocystin	20	Roquefortine	10
Atpenin A5	10	15-AcetylDON	10.4	Brevicompanin B	10	AOD	10
Asperlactone	20	Neosolaniol	8			AAL TA- Toxin	10
Calphostin C	2.5	Deepxoxy-DON	4	**Substances [MIX 15]**	**conc.**	**Substances [MIX 16]**	**conc.**
Aspyrone	20	DON-Glucosid	2.7	Citreoviridin	10.2	Curvularin	20
Pyripyropene A	10	Ochratoxin B	0.7	Malformin C	20	Territrem B	20
Equisetin	10	Ochratoxin A	3.3	16-Ketoaspergillimide	20	Aspinonene	20
Stachybotrylactam	10	T2-Triol	3.4	Aspergillimide	20	Decarestrictine	20
Viridicatin	20	T2-Tetraol	3.2	Tenuazonic acid	14.3	Cycloaspeptide	20
**Substances [MIX 13]**	**conc.**	**Substances [MIX 14]**	**conc.**	NG 012	20	Tetracycline	20
Methysergide	2.8	Ustiloxin A	10	Neoxalin	20	Chloramphenicol	20
Ergocryptinin	2.8	Ustiloxin B	10	Geodin	20	Oxaspirodion	20
Ergocorninine	2.8	Ustiloxin D	10	Pyreophorol	20	Cycloheximide	20
Erginine	2.8	Erythromycin	9.5	Desferrioxamine E	20	Asperloxine A	20
Ergosinin	2.8	Fusidic acid	10	**Substances [MIX 19]**	**conc.**	**Substances [MIX 20]**	**conc.**
Ergometrinin	1.8	Amphotericin	15	Rugulosin	20	Citromycetin	32
Ergocristinin	2.8	Bacitracin	15	Penigequinolone A	10	Cyclopeptin	20
Ergotaminin	2.8	Neomycin	10	Terphenyllin	20	3-Methylviridicatin	10
		Vancomycin	10	Cycloechinullin	10	Fusaproliferin	30
**Substances [MIX 17]**	**conc.**	**Substances [MIX 18]**	**conc.**	Ophiobolin B	30	Marcfortine A	4
Nigericin	1.2	K 252b	0.9	Deoxybrevianamid E	30	Clamydosporol	20
Anisomycin	1.4	Myriocin	0.8	Aspercolorin	10	Trichodermol	40
Nonactin	0.5	Ionomycin	0.9	Nornidulin	10	Thiolutin	6.4
Oligomycin A	1.7	Oligomycin	1.6	Nidulin	10	Fusarielin A	20
FK 506	1.3	Puromycin	1	Fulvic acid	30	Aureobasidin	100
Actinomycin D	1.4	Mitomycin	4.9	Lolitrem	0.1	Dechlorogriseofulvin	20
Cerulenin	5.1	Rapamycin	0.8	A23187	5		
Radiciol	2.1	Geldanamycin	1.2				
**Substances [MIX 21]**	**conc.**	**Substances [MIX 22]**	**conc.**	**Substances [MIX 23]**	**conc.**		
Mithramycin C	2.1	Moniliformin	69.8	Fumonisin B1	35.1		
Staursporine	1.4	Z4G	47.2	Fumonisin B2	35.9		
Valinomycin	2	Alamethicin	37.7	Fumonisin B3	4.5		
Trichostatin	1.6	Z4S	0.6	hydrolysed FB1	13.3		
Ascomycin	1.7			alpha OTA	1.7		
Bafilomycin	2						
K252a	2.2						

**Table 4:** Identification and specification of used test kits.

Test Kit Identification	Cutoff Level [µg/kg]
test kit A	4
test kit B	10
test kit C	20
